# de crusta lactea infantum & ejusdem specifico remedio dissertatio, quam scientiarum artium atque literarum academia quæ Lugduni in Galliis est altero duplici præmio coronavit die 3 Decemb. 1776

**Published:** 1781-09

**Authors:** 


IV. Caroli Strack, M. D. *in Univerſitate Mogunt. Inſtit. Med. Profeſſ.* &c. *de cruſta lactea infantum & ejuſdem ſpecifico remedio diſſertatio, quam ſcientiarum artium atque literarum academia quæ Lugduni in Galliis eſt altero duplici præmio coronavit die* 3 *Decemb.* 1776. 8vo. Francf. ad Mœnum, 1779. p. 61.

THE diſeaſe which is the ſubject of this diſſertation generally attacks children at the breaſt, and begins to diſappear ſoon after they are weaned. In ſome however it makes its appearance at a later period and continues even to the ſixth year, and our author ſpeaks of a child who had a return of the diſorder in his fourth year. It commonly begins on the cheeks, with an eruption of pimples filled with a limpid fluid. As the pimples crack, this fluid hardens, and by degrees forms a thick cruſt of a reddiſh yellow colour. As the diſeaſe advances, the ſkin underneath it ſwells and hardens, and at length the glands of the neck, and ſometimes, though rarely, the parotid glands begin to be affected.

In ſome children this ſcurf ſpreads behind the ears, and down to the chin, disfiguring the whole face, the eye-lids excepted. In ſome it is attended with a running at the ears, and in others, though leſs frequently, with ophthalmia. Now and then it affects the neck, breaſt, abdomen, and extremities.

Our author acknowledges his inability to aſcertain the real cauſe of this diſorder. He ſuppoſes it, however, to be contagious and even hereditary. He has obſerved that children, whoſe mothers have been ſubject to it in their infancy, are more liable to it than others though ſuckled by other women, and that nurſes who have themſelves been affected with it ſeldom fail to communicate it to the children they ſuckle.

Our author refutes the commonly-received notion, that this diſorder is a ſecurity from the ſmall pox. He obſerves, that if it attacks the glands or is repelled by topical applications it is ſometimes productive of the worſt effects, and he likewiſe conſiders the ſcurf's not breaking, ſo as to afford a ſufficient outlet to the matter, as an unfavourable circumſtance, it being apt in ſuch caſes to attack the glands of the meſentery.

Dr. Strack remarks of this diſeaſe, that the conſtant itching that attends it deprives the patients of ſleep, and of courſe emaciates them. When the genitals happen to be affected the urine excoriates the parts and adds to the complaint. If left to nature it generally diſappears in about ſix months. In ſome however it is of much longer duration. He has obſerved of thoſe who have been relieved by nature, without the interpoſition of remedies, that the urine has ſmelt like that of cats. The ſooner this happens the ſooner the ſcurf dries off; but when the urine retains its uſual odour the diſeaſe is generally of long continuance, and as there is always danger of its terminating in tabes meſenterica or ſome other fatal complaint, it will ſeldom be prudent to leave it to itſelf.

The ſpecific recommended by our author for the cure of this diſorder is the *viola tricolor* Linn. A handful of the freſh or half a drachm of the dried leaves of this plant are to be boiled in half a pint of cow's milk, which is afterwards to be ſtrained for uſe. This doſe is to be repeated every morning and evening. He obſerves, that when it has been adminiſtered eight days in this manner, the eruption uſually increaſes conſiderably, even in thoſe who had it only in a ſlight degree previous to the ufe of this remedy, the urine at the fame time acquiring the fmell juft now fpoken of. When the medicine has been continued a fortnight the fcurf commonly begins to fall off in large fcales, and the fkin underneath it appears clean. In order to prevent a relapfe we are directed to perfevere in the ufe of the remedy till the fkin has refumed its natural appearance and the urine ceafes to have any particular fmell. A variety of cafes are related in which this medicine is faid to have fucceeded; and the author afferts, that during the twenty years he has prefcribed it, he has hardly ever known it to fail.——Its fimplicity will probably recommend it to the notice of medical practitioners, and we foon hope to hear that its efficacy has been put to the teft in this country.

